# Point Mutations in c-Myc Uncouple Neoplastic Transformation from Multiple Other Phenotypes in Rat Fibroblasts

**DOI:** 10.1371/journal.pone.0013717

**Published:** 2010-10-28

**Authors:** J. Anthony Graves, Kristi Rothermund, Tao Wang, Wei Qian, Bennett Van Houten, Edward V. Prochownik

**Affiliations:** 1 Division of Hematology/Oncology, Department of Pediatrics, Children's Hospital of Pittsburgh of The University of Pittsburgh Medical Center, Pittsburgh, Pennsylvania, United States of America; 2 The University of Pittsburgh Cancer Institute, Pittsburgh, Pennsylvania, United States of America; 3 The Department of Pharmacology and Chemical Biology, The University of Pittsburgh Medical Center, Pittsburgh, Pennsylvania, United States of America; 4 The Department of Microbiology and Molecular Genetics, The University of Pittsburgh Medical Center, Pittsburgh, Pennsylvania, United States of America; University of Illinois at Chicago, United States of America

## Abstract

Deregulation of c-Myc (Myc) occurs in many cancers. In addition to transforming various cell types, Myc also influences additional transformation-associated cellular phenotypes including proliferation, survival, genomic instability, reactive oxygen species production, and metabolism. Although Myc is wild type in most cancers (wtMyc), it occasionally acquires point mutations in certain lymphomas. Some of these mutations confer a survival advantage despite partially attenuating proliferation and transformation. Here, we have evaluated four naturally-occurring or synthetic point mutations of Myc for their ability to affect these phenotypes, as well as to promote genomic instability, to generate reactive oxygen species and to up-regulate aerobic glycolysis and oxidative phosphorylation. Our findings indicate that many of these phenotypes are genetically and functionally independent of one another and are not necessary for transformation. Specifically, the higher rate of glucose metabolism known to be associated with wtMyc deregulation was found to be independent of transformation. One mutation (Q131R) was greatly impaired for nearly all of the studied Myc phenotypes, yet was able to retain some ability to transform. These findings indicate that, while the Myc phenotypes examined here make additive contributions to transformation, none, with the possible exception of increased reliance on extracellular glutamine for survival, are necessary for achieving this state.

## Introduction

Deregulation of the c-Myc oncoprotein (hereafter Myc) occurs in many human cancers, generally as a consequence of *MYC* gene amplification or its aberrant transcriptional regulation [1]. In certain lymphomas, Myc over-expression results from a chromosomal translocation of *MYC* into an immunoglobulin gene locus, which suffices to drive the high-level expression of the proto-oncogene in the pre-B or B cell environment [1], [2].

Although the Myc protein sequence remains unaltered in most cancers, exceptions occur in the above-cited lymphomas, where over half contain recurrent and often multiple Myc point mutations, which are generally confined to the N-terminal transcriptional regulatory domain (TRD). These commonly cluster around Thr_58_ and Ser_62_ whose phosphorylation status greatly affects protein stability, transactivation and transformation [3], [4], [5]. Less frequent mutations involve an evolutionarily conserved 15–20 residue segment of the TRD known as Myc Box II (MBII) [6], [7], [8], [9], which contributes importantly to the transforming function of Myc and is a site of several significant protein-protein interactions that influence transformation [10], [11], [12], [13]. Burkitt's lymphoma-associated point mutations within MBII have been shown to reduce the ability of Myc to promote apoptosis while concurrently attenuating its transforming capacity and/or ability to stimulate growth [14], [15], [16]. Enhanced survival of Myc overexpressing cells thus seems to be a property that is highly selected for during the course of tumor evolution and may be more important than transformation itself. The exact contribution of each point mutation to these phenotypes is unclear, however, as they often occur in the context of other point mutations.

A hallmark of many cancer cells is a high rate of glycolysis, even in the face of sufficiently high oxygen levels to support oxidative phosphorylation (OXPHOS). Defined as the “Warburg effect”, this was initially attributed to a functional impairment of OXPHOS and/or defective mitochondrial biogenesis [17]. More recently, it has been viewed as allowing a reprogramming of cellular energy metabolism whereby TCA cycle intermediates can be redirected toward the enhanced synthesis of the amino acids, fatty acids, and nucleotides needed to support the more robust growth and proliferative demands of the transformed state [18]. Indeed, Myc over-expression actually leads to increased mitochondrial biogenesis and the induction of select enzymes of the TCA pathway [19], [20]. As a result of this shift in metabolic balance, and because the glycolytic pathway is a less efficient ATP energy source, many transformed cells seem to be extremely dependent on external glucose supplies (i.e.- they become glucose “addicted”)[21]. Consistent with this, many cancer cells express high levels of glycolytic enzymes [22] and the genes encoding a number of these are also Myc-regulated [20], [23]. It has been proposed that the ability of Myc to alter metabolism in this manner is an essential feature of its ability to promote cell cycle entry [24], although a functional metabolic link to other cellular behaviors remains to be established. Together, these metabolic changes may also explain the profound reliance of cancer cells on exogenous glutamine. Because this amino acid is transported into the mitochondria and subsequently enters the TCA cycle as glutamate, it has been proposed that this provides the intermediates for and facilitates the generation of biosynthetic pathway precursors [18], [25].

The increased aerobic glycolysis that defines the Warburg effect emerges gradually and in response to the step-wise activation of oncogenes and loss of the TP53 tumor suppressor pathway that contribute to the fully transformed state [26], [27]. However, it remains unclear to what extent this and other metabolic changes specifically contribute to transformation. Using defined Myc point mutants that retain only certain Myc functions, we have asked how the loss of specific phenotypes affects others, particularly those pertaining to metabolism and transformation. We report here that many Myc phenotypes, including the Warburg effect and enhanced OXPHOS, can be genetically uncoupled. Moreover, none of the specific phenotypes examined are required for transformation, although they do appear to influence the efficiency with which transformation occurs.

## Results

### Expression of Myc point mutants

**Figure 1:**
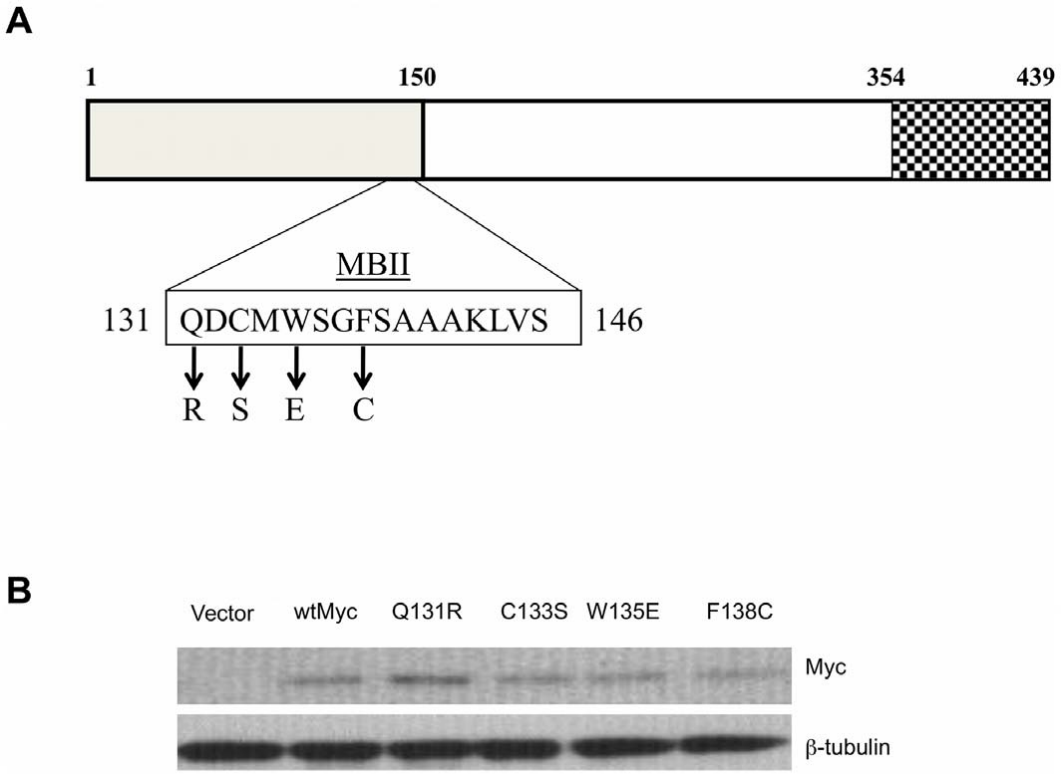
Expression of Myc mutants in Rat1a cells. (**a**) Diagram of the Myc protein. The approximately 150 residue TRD is shaded, with an expanded MBII domain and relevant amino acids substitutions depicted below the diagram. The basic-helix-loop-helix dimerization domain at the extreme C-terminus of the protein is indicated by the checkered box. (**b**) Each of the indicated mutations, along with wtMyc was expressed in Rat1a fibroblasts following lentiviral-mediated transduction. An empty lentiviral vector infection served as a negative control. Pooled, blasticidin-resistant colonies were subjected to western analysis for either Myc or β-tubulin, which served as a loading control. The monoclonal antibody used to detect human Myc proteins had some cross reactivity with endogenous rat Myc.

Previous studies have shown that several naturally-occurring and synthetic MBII point mutations affect the ability of Myc to transform and/or promote proliferation and apoptosis. However, some of these proteins contained multiple TRD mutations, were assessed only for a limited number of phenotypes, or were evaluated in different cell types that complicated or precluded cross-study comparisons [10], [14], [15], [28]. We therefore re-examined these properties, as well as several additional ones, in the same cell line for four individual point mutants (Figure 1a). The Q131R and C133S constructs alter residues that have been targeted in previous studies [15]. The F138C mutant has been found to occur naturally in Burkitt's lymphoma, although it typically appears in combination with other mutations [5], [28]. The W135E mutant has been identified as having a reduced transformation capacity [15] and has been speculated to be deficient it its ability to interact with other proteins such as TRAAP [29]. These human proteins were expressed in Rat1a fibroblasts, because this cell background has been routinely used to evaluate numerous Myc phenotypes, including anchorage-independent transformation and in vivo tumorigenesis [14], [15], [30], [31]. Following high-efficiency lentiviral-mediated transduction, surviving clones were pooled and immediately examined for the expression of their respective proteins. As seen in Figure 1b, all four mutants were expressed at similar levels and were comparable to the expression of the wild-type Myc (wtMyc) protein.

### Mutant Myc proteins differentially affect proliferation and apoptosis

**Figure 2:**
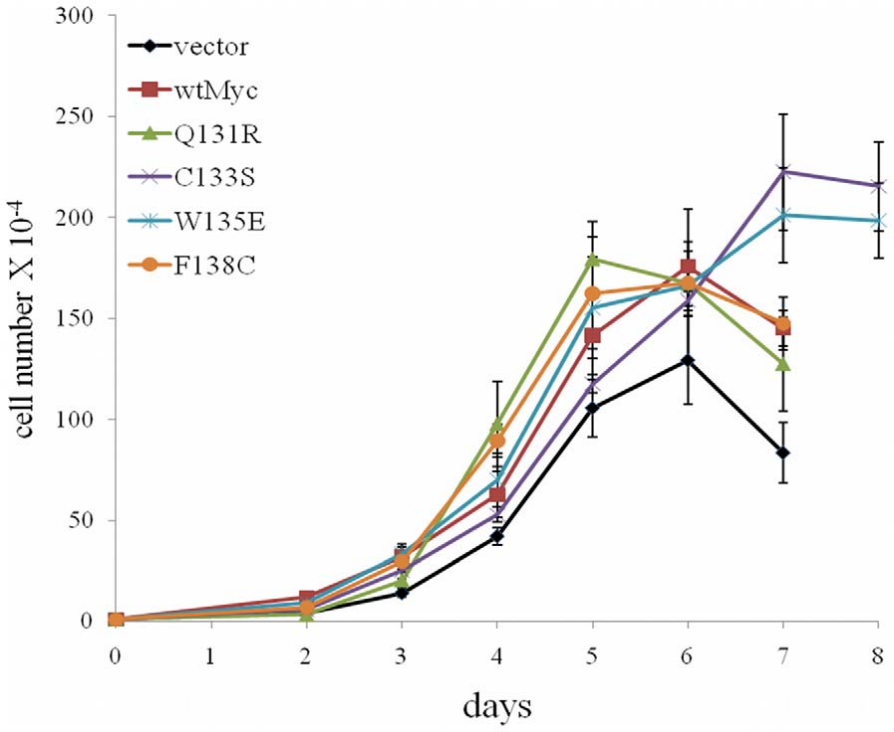
Effect of Myc point mutations on proliferation. The indicated Rat1a cell lines were seeded at 10^4^ cells/well in 12 well plates and allowed to attach in medium containing 10% FBS. At the indicated times, the total cell number in triplicate wells was determined. The points shown represent the average number of cells/well +/− 1 standard error (SE). The experiment was repeated on at least three occasions with similar results. Only adherent cells were counted. The number of cells at the latter time points may be affected by contact inhibition or cell loss due to overgrowth.

**Figure 3:**
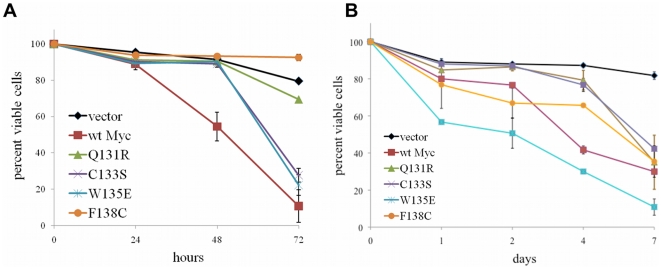
Cell death mediated by Myc proteins. The indicated Rat1a cell lines were seeded into 12 well pates at 10^5^ cells/well and allowed to achieve approximately 80% confluency. At the indicated times, viable cell numbers in triplicate plates were determined by flow cytometry using an Annexin V-Propidium Iodide staining protocol as described in Materials and Methods. The results represent the average amount of staining for the triplicate samples +/− 1 SE. The experiment was repeated on at least three occasions with similar results. (**a**) Serum withdrawal. The cells were washed in PBS and incubated in serum-free medium for the remainder of the study. (**b**) Glutamine withdrawal. The cells were washed in PBS and incubated in glutamine-free medium for the remainder of the study.

Myc confers a proliferative advantage when over-expressed in various cell backgrounds including Rat1a cells [32]. We therefore compared the growth rates of the six Rat1a cell lines depicted in Figure 1 under non-limiting growth conditions (Figure 2). When viable, adherent cells were enumerated, it was observed that all MBII mutants and wtMyc cells grew modestly faster than did the vector control line. C133S and W135E cells also attained approximately a 1.5-2-fold higher final density and remained viable longer. We next asked whether any of the mutations altered the well-known ability of Myc over-expression to sensitize cells to pro-apoptotic stimuli [15], [33], [34], [35]. We therefore grew each of the cell lines to approximately 80% confluency under non-limiting conditions, then changed to media lacking either serum (Figure 3a) or glutamine (Figure 3b), and subsequently assessed viability. As seen in Figure 3a, wtMyc cells showed the expected high sensitivity to serum deprivation as manifested by an approximately 9-fold higher fraction of apoptotic cells at 72 hours when compared to vector cells. W135E and C133S cells were almost as sensitive as wtMyc cells to serum withdrawal, whereas Q131R cells and F138C cells, like vector cells, were resistant.

The cell death induced by glutamine withdrawal did not produce as robust of a response as did serum deprivation, taking approximately one week to observe significant cell death. Whereas the control cells were mostly resistant to death under these conditions, wtMyc, Q131R, C133S and F138C all showed similar ultimate levels of demise, although the rates of death were somewhat slower for all the mutants. In contrast, W135E showed a modestly accelerated rate of apoptosis. From these studies we conclude that Myc mutants are differentially responsive to serum and glutamine deprivation. Moreover, while at least two mutants (Q131R and F138C) were unable to impart a pro-apoptotic response to serum removal, none entirely abolished the response to glutamine depletion.

### Mutant Myc proteins differentially affect genomic instability

Myc affects genomic stability at multiple levels. For example, Myc over-expression, particularly when coupled with TP53 dysfunction, can lead to the stochastic accumulation of tetraploid cells, which are particularly prone to further chromosomal instability [36], [37], [38]. Myc deregulation also induces high levels of ROS, which can induce double-stranded DNA breaks and point mutations [39], [40]. It has been proposed that the potency of Myc as an oncoprotein originates from its unrelenting assault on genomic integrity and serves as a driver of tumor cell evolution [41]. However, the relationships among these processes and those investigated above have not been formally tested.

**Figure 4:**
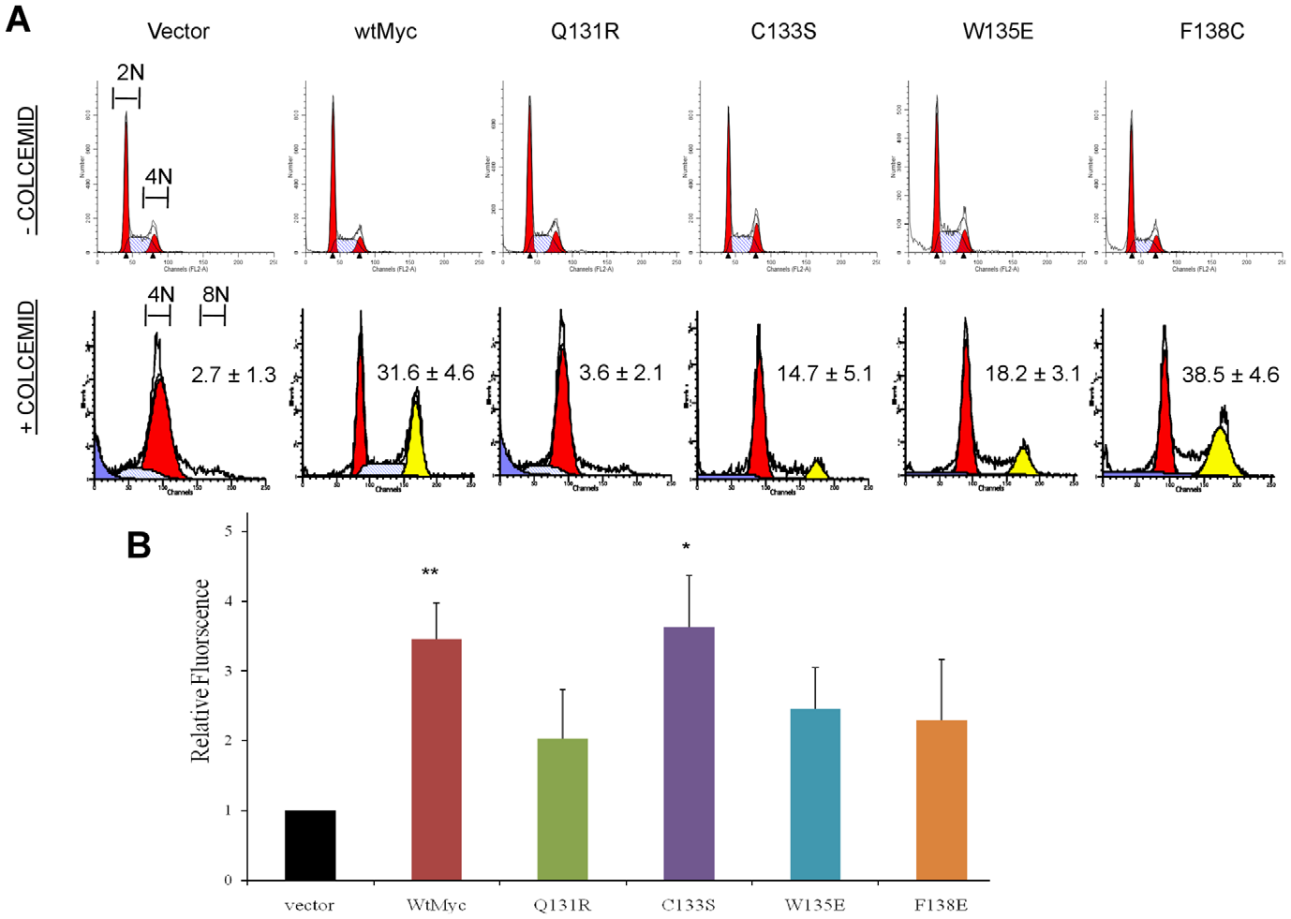
GI induced by Myc proteins. (**a**) Induction of tetraploidy. Each of the depicted cell lines showed indistinguishable cell cycle parameters during logarithmic growth in the absence of colcemid (top row). Treatment of vector cells with colcemid resulted in the expected accumulation of G2/M-arrest cells containing a 4N DNA content, whereas a significant fraction of wtMyc cells accumulated tetraploid DNA content (8N bottom row) [28], [58]. Note the varying degrees of tetraploidy induction in all mutant cell lines except Q131R. (**b**) Induction of ROS. Each of the cell lines was exposed to CM-H_2_-DCFDA and was then assessed by flow cytometry. Note the >3-fold higher levels of ROS in wtMyc cells compared to vector control cells as previously described [33], [39] and that only C133S cells were able to reproduce this effect. p-values were calculated by a two-tailed t-test in Microsoft Excel when compared to vector cells: (*) p≤0.05; (**) p≤0.005.

To assess the ability of Myc proteins to promote genomic instability (GI), we first exposed each of the above six cell lines to colcemid and then quantified the resulting fraction of tetraploid cells by flow cytometry. As seen in Figure 4a, vector cells routinely arrested in G2/M following short-term exposure to the drug whereas a substantial fraction of wtMyc cells became tetraploid [37], [38]. C133S, W135E, and F138C cells recapitulated this to varying degrees, whereas Q131R cells failed to show any evidence of tetraploid formation.

We next quantified ROS production in these cells using the fluorescent activation of CM-H_2_-DCFDA as a surrogate marker [39], [40], [42]. As expected [40], [42], wtMyc cells showed significantly higher baseline levels of ROS than did vector cells. In contrast, only C133S cells were able to generate comparable levels of ROS (Figure 4b). From these and the foregoing studies, we conclude that Myc mutations differentially affect tetraploidy and ROS induction and that these two properties are genetically separable.

### Real-time assessment of OXPHOS and glycolysis

**Figure 5:**
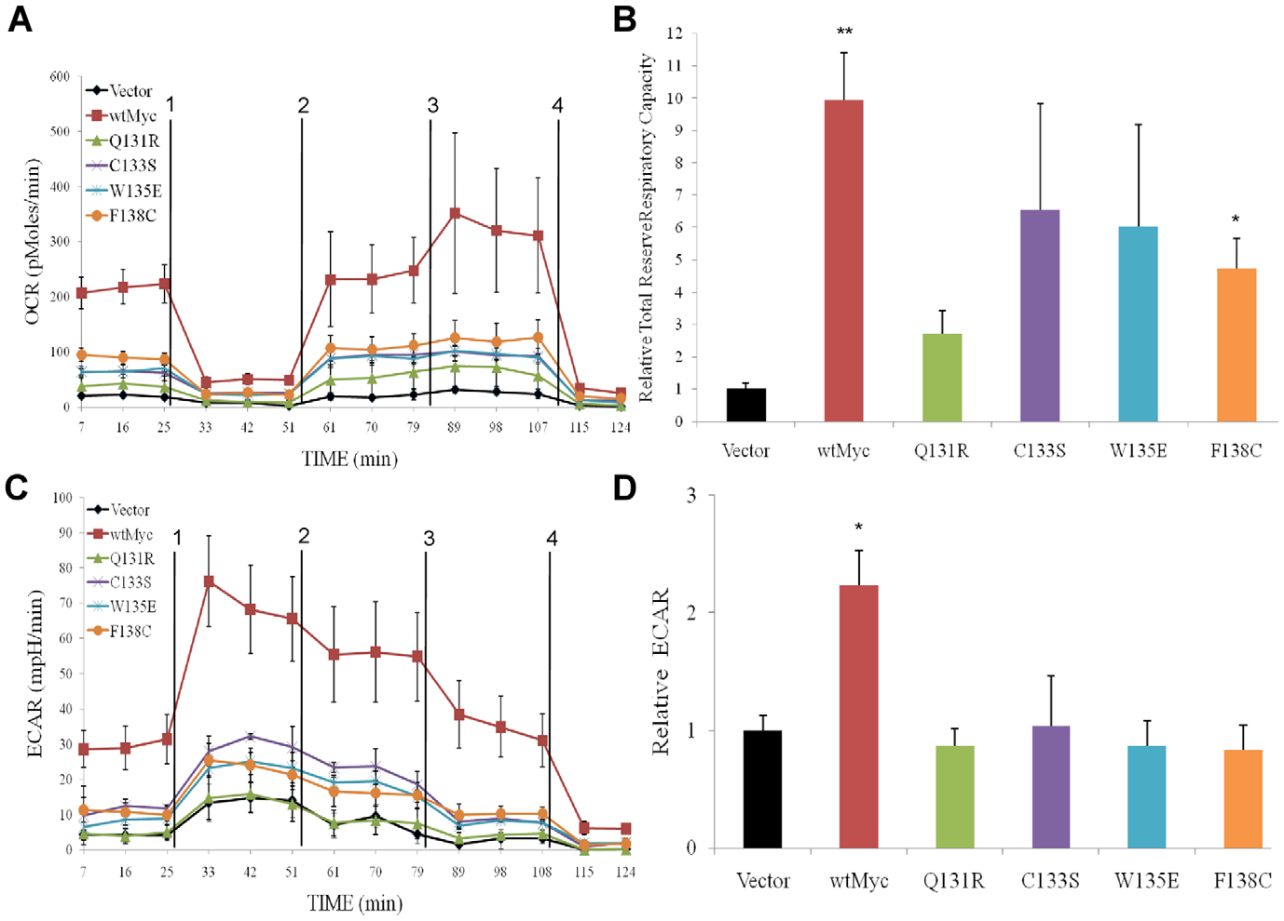
OCR and ECAR in Rat1a cells expressing human Myc point mutants. (**a**) A Seahorse Bioscience XF24 Extracellular Flux Analyzer was used for the real-time determination of metabolism. OCR (OXPHOS) was expressed as a function of time. Each inhibitor as described in Results was injected at the times indicated by the vertical lines (injections 1–4). A typical experiment, performed in triplicate wells is shown. The experiment was repeated on at least three occasions with similar results. (**b**) Areas under the curve (AUC) were calculated with the software provided by the manufacturer and used to compare the various cell lines' total reserve respiratory capacity relative to that of vector control cells, which are arbitrarily set at 1. The average of three to five individual experiments, where each cell line was measured at least in triplicate is displayed +/− 1 SE. p-values of the comparisons with vector control cells were calculated by a two-tailed t-test in Microsoft Excel: (*) p≤0.05; (**) 0.01. This graph represents the AUCs for the time point just prior to injection 2 until the time point just before injection 4. This represents the total reserve respiratory capacity of each cell line. (**c**) ECARs (glycolysis) were expressed as a function of time. The vertical lines represent the same injections as previously described. The experiment was repeated on at least three occasions with similar results. (**d**) AUCs for the time point just prior to injection 1 to the time point just prior to injection 2 were calculated. This is the best representation of the glycolytic potential of these strains. p-values were calculated and represented as described for (**b**).

An XF24 Extracellular Flux Analyzer (Seahorse Bioscience) was used to obtain real-time determinations of parameters pertaining to OXPHOS and glycolysis. OXPHOS is represented by the oxygen consumption rate (OCR) [43], which is expressed as pmol per minute as oxygen undergoes a four-electron reduction to water at complex IV. As represented in Figure 5a, and as subsequently confirmed in multiple experiments, baseline OCR (measured between 0–25 min) was consistently 10 fold higher in wtMyc cells than in vector control cells. These results confirmed previous reports documenting Myc-mediated enhancement of OXPHOS [24], [44], [45], [46]. In three cases (C133S, W135E, and F138C) the baseline OCRs were between those of wtMyc and vector control cells, suggesting that these mutations reduced, but did not eliminate, the increase in OXPHOS. In contrast, baseline OCR for Q131R cells was identical to that of vector control cells.

Following these initial determinations, all cell lines were sequentially and simultaneously exposed to four different inhibitors to yield a profile of bio-energetic capacity. Oligomycin (injection 1) prevents the flow of protons from the mitochondrial intermembrane space back into the matrix through complex V. This causes a buildup of protons and a subsequent decrease in the ability of electrons to flow down the electron transport chain. The result is a large decrease in OCR and is indicative of marked inhibition of OXPHOS (Figure 5a). Cyanide *p*-trifluoromethoxy-phenylhydrazone (FCCP; injection 2) causes protons on the outside of the inner mitochondrial membrane to be carried across to the basic matrix, allowing both electron flow to be re-established and oxygen consumption to resume at levels equal to or exceeding those of the original baseline despite the persistent oligomycin-induced block of complex V. This is believed to be a measure of “spare respiration capacity (sRC)” [47], [48]. Following FCCP injection, wtMyc cells showed the largest sRC whereas vector control cells and Q131R cells showed the smallest. The remaining three Myc mutant cell lines showed intermediate sRCs. 2-deoxy-D-glucose (2-DG) was then added to inhibit glycolysis (injection 3). In all cell lines, this resulted in a further increase in OCR. This increase, in combination with the sRC, represents the “total reserve respiratory capacity” (tRRC) of the cell [47], [48]. wtMyc displayed the largest tRRC, which was 10-fold higher than vector control cells. All Myc mutants had comparatively less tRRC, and only F138C was significantly elevated relative to the control (Figure 5b). Finally, the injection of rotenone (injection 4), which inhibits complex I, resulted in the complete cessation of electron flow and oxygen consumption in all cell lines.

Concurrently measured with OCR was the extracellular acidification rate (ECAR), expressed as mpH units per minute, which is a measure of the glycolytic conversion of glucose to lactate. Basal ECARs for wtMyc cells were approximately 7-fold higher when compared to vector control cells (Figure 5c, 0–25 min), supporting previous studies showing that Myc over-expression increases glycolysis as well as OXPHOS [24]. All four Myc mutants displayed reduced ECARs. Three to five replicate experiments showed that none of the baseline ECAR values were significantly different from vector control cell values.

As had been done for OCR, ECAR was measured as the previously described pharmacologic inhibitors were added. All cell lines showed an increase in ECAR as glycolysis became the major energy source following the poisoning of OXPHOS by oligomycin (injection 1) and the resumption of electron flow following the addition of FCCP (injection 2). The area under the curve between these two injection points represents a measure of glycolytic potential [47], [48] and only wtMyc cells showed elevated ECAR relative to vector control cells at this stage (2.2 fold difference, Figure 5d). Upon the resumption of electron flow and oxygen consumption following FCCP addition (injection 2), all cell lines showed a reduction in ECAR values, which underwent the expected further decline following the addition of 2-DG (injection 3). From these studies, we conclude that the Myc mutants, particularly Q131R, were all compromised with respect to their ability to increase OXPHOS and glycolysis but that in all cases, each pathway retained the ability to respond in an appropriate and reciprocal manner upon inhibition of the other pathway.

### Mutant Myc proteins retain competency for transformation

**Figure 6:**
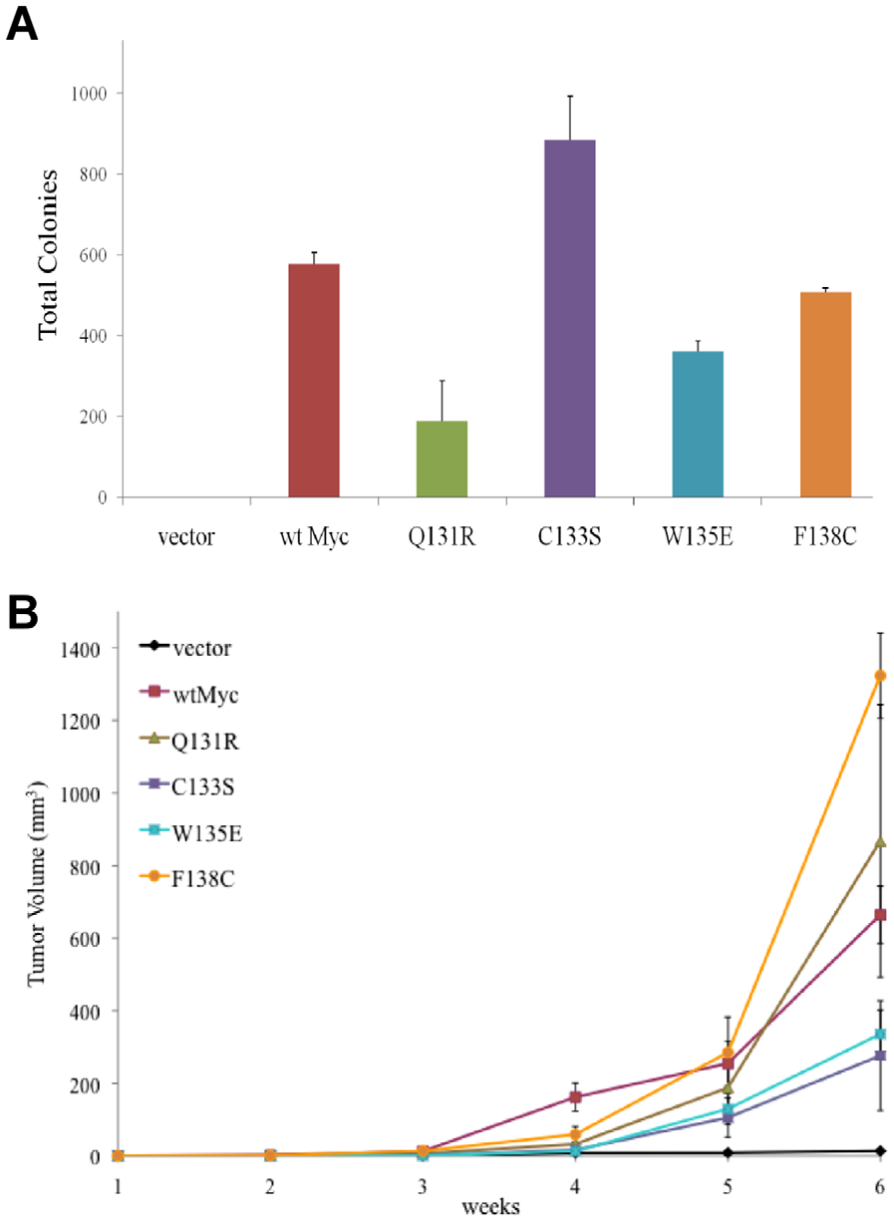
Effect of Myc point mutations on transformation. (**a**) In vitro transformation. Anchorage-independent clonogenic growth in soft agar was assessed as previously described [54], [56]. 12–14 days after plating, the total number of macroscopically visible colonies on each plate was quantified. Numbers shown represent the average number of colonies seen in triplicate cultures +/− 1 SE. p-values of the comparisons with vector control cells were calculated by a two-tailed t-test in Microsoft Excel: (**) p≤0.01; (***) ≤0.005. (**b**) In vivo transformation. 5×10^6^ of the indicated Rat1a cell lines were inoculated subcutaneously into the flanks of nude mice. Each inoculum was performed in triplicate and tumor sizes were evaluated weekly. The data shown represents two individually repeated experiments. Averages tumor sizes [52] +/− 1 SE are depicted as a function of time.

Given that each of the four mutant Myc proteins was in some way defective for one or more Myc phenotypes, we next asked how they affected transformation. We were particularly interested in Q131R, which was most restricted in its ability to confer any of the non-transforming Myc phenotypes thus far examined. As seen in Figure 6a, three of the point mutant cell lines (Q131R, W135E, and F138C) showed a modest to moderate (approximately 30–70%) reduction in anchorage-independent clonogenic growth compared to wtMyc cells whereas C133S cells were somewhat more efficient. These findings were corroborated in tumor xenograft assays, which showed that all of the mutant cell lines were capable of tumorigenic growth, although lag times were prolonged and did not correlate well with in vitro clonogenicity assays (Figure 6b). Most notably, tumors arising from the inoculation of Q131R cells grew at rates indistinguishable from those formed by wtMyc cells.

### *In vivo* measurement of tumor metabolism

**Figure 7:**
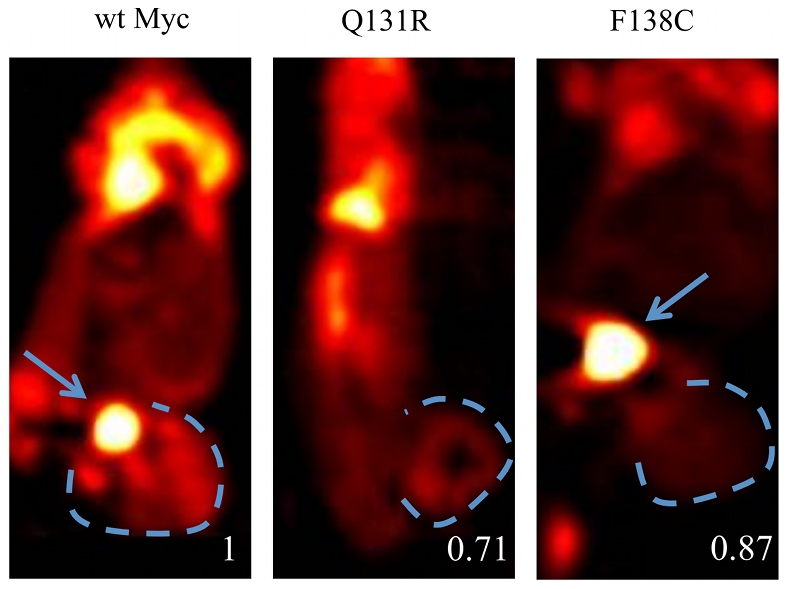
^18^F-2DG imaging by PET. Duplicate tumor-bearing mice inoculated with wtMyc, Q131R, or F138C cells were imaged by PET following injection of ^18^F-labeled 2DG. Dotted lines indicate tumor boundaries. Arrows indicate the urinary bladder, where the isotope is expectedly the most concentrated. The average relative SUV is indicated with the value for wtMyc set at 1.

To determine whether the metabolic defects described above may have been confined only to the in vitro setting, we used positron emissions tomography to compare the uptake of ^18^F-2-DG in tumor xenografts arising from wtMyc, Q131R and F138C cells. As shown in Figure 7 the latter tumors were only slightly less metabolically active than the former. From these studies, we conclude that the decrease in metabolic up-regulation seen in Q131R and F138C cells is a consistent finding that is independent of the tumor cell environment.

## Discussion

Myc deregulation in many cell types, including Rat1a fibroblasts, affects multiple phenotypes, including proliferation, apoptosis, and GI [14], [15], [16], [19]. More recently, Myc deregulation has been implicated in altering cellular metabolism, particularly by inducing both the Warburg effect and a reliance on exogenous glutamine [18], [25], [49]. Although these properties are likely intertwined with transformation at some level, their individual contributions remain largely unknown. Previous work, however, has shown that some of these phenotypes can be severely compromised without exerting significant effects on transformation. For example, cells expressing certain Burkitt's lymphoma-associated point mutants are more resistant to pro-apoptotic stimuli and less responsive to proliferative signals than are those expressing wtMyc [14], [15], [16]. This has suggested that such mutants are selected for because they impart a relative survival advantage even if they are transformation deficient [10], [14], [15]. We have characterized a set of Myc mutants in an attempt to better define the roles of the various phenotypes on tumor growth. We individually mutated four residues (Q131, C133, W135, and F138), which have been previously shown either to occur naturally (albeit rarely) in Burkitt's lymphomas and/or to interfere in experimental systems with the transformation potential of Myc or its ability to interact with transcriptional co-regulators [5], [12], [15], [28], [42]. This represents the first study in which mutants of this type have been investigated for their ability to influence metabolism. We have chosen to express these mutants in Rat1a fibroblasts as they have been repeatedly used to assay a variety of phenotypes including cell transformation following only the over-expression of wtMyc [14], [15], [30], [31], and therefore represent the only system in which the specific effects on these various phenotypes could be thoroughly studied in an isogenic background.

Increased OXPHOS has been shown to be important for cell cycle entry and is commonly linked to transformation [24]. Aerobic glycolysis, or the so-called Warburg effect, is also frequently observed in cancer cells although it is not invariably associated with transformation. However, it may become more prominent when the cells are grown as tumors or metabolically stressed by hypoxia [18], [23], [24], [25], [50]. It does appear, however, that virtually all cancer cells increase their glucose metabolism via some combination of increased OXPHOS and aerobic glycolysis [19], [50].

The use of pharmacologic agents to inhibit OXPHOS and glycolysis at various steps indicated that appropriate and reciprocal changes in the other pathway occurred in each case (Figure 5). These findings do not support the idea that qualitative defects in specific TCA cycle enzymatic pathways account for the switch from OXPHOS to aerobic glycolysis seen in the majority of cancers [20]. Rather, they are consistent with the notion that the enhanced metabolism of Myc-deregulated tumor cells is much more commonly due to increases in the otherwise functionally normal enzymes comprising these two metabolic pathways [18], [25]. Such quantitative changes would seem to be the best suited to addressing the demands of the transformed cell for various TCA cycle-derived anabolic precursors [18], [24], [51] while simultaneously providing the flexibility needed to increase glycolysis in response to the hypoxic tumor microenvironment. However, our results also indicate that transformation per se requires neither increased OXPHOS nor induction of the Warburg effect, although it is possible that these changes contribute to the efficiency with which transformed cells can grow as anchorage-independent colonies or as xenografts in vivo (Figure 6).

We are unable to say unequivocally that transformation and glutamine addiction can be dissociated from one another given that none of the mutants tested entirely relieved the cellular reliance on glutamine. Our analyses do however, allow us to conclude that the normal levels of OXPHOS and glycolysis seen in several of the cell lines are not necessarily associated with normalization of glutamine dependency. These studies again suggest these processes are under distinct forms of genetic regulation as has been shown for other Myc phenotypes [15].

High levels of potentially mutagenic ROS have been proposed to arise from an increased flux of electrons through the electron transport chain as a result of the more robust OXPHOS of transformed cells [20], [40]. However, we noted discordance between ROS production and OXPHOS in three of the four Myc mutants (Q131R, W135E, and F138C). Indeed, a comparison of C133S and F138C, both of which showed equivalent reductions in OXPHOS levels relative to wtMyc indicated that only the former was able to enhance ROS levels in Rat1a cells (Figure 4b). Taken together, these findings demonstrate that, while much of the Myc-mediated ROS induction may well have a mitochondrial origin, a significant fraction appears to originate elsewhere, perhaps as a consequence of the increased sensitivity of Myc-regulated proliferation-associated or other signaling pathways [52]. They also indicate that elevated OXPHOS does not invariably lead to the generation of high levels of ROS. Additional studies will be needed to define the mitochondrial and non-mitochondrial sources of ROS.

In summary, our findings indicate that single point mutations in of the MBII domain of Myc can genetically uncouple transformation from several of the phenotypes that contribute to and are closely associated with the transformed state. Additionally, several of these phenotypes are genetically separable from each other. The number and the degree to which these phenotypes are affected are variable and dependent upon the specific point mutation. Specifically, the dispensability of these various Myc-regulated behaviors for transformation was most conspicuously exemplified by Q131R cells, which were lacking in most of the studied phenotypes, yet were still able to grow as anchorage-independent colonies and form tumors at nearly the same rate as wtMyc cells. The multiple Myc phenotypes examined here, including the metabolic ones, thus appear to affect the efficiency with which cells can be transformed but are to a large degree dispensable. Most significantly, we have shown that these phenotypes include the Warburg effect and that neither increased aerobic glycolysis nor particularly elevated OXPHOS are absolutely necessary for achieving a transformed state.

## Materials and Methods

### Construction of Myc mutants

All human Myc proteins were expressed in the pLenti-V5-TOPO-Blast vector (Invitrogen, Inc. Carlsbad, CA) as non-epitope-tagged full-length proteins. The vector encoding wild-type Myc was used as a template for oligonucleotide directed point mutagenesis utilizing a QuickChange II kit according to the directions of the supplier (Stratagene, Inc. LaJolla CA). All lentiviral vectors were propagated in the Stbl3 E. coli strain (Invitrogen) and mutations were confirmed by automated DNA sequencing.

### Cell Culture and lentiviral transductions

All lentiviral work was approved by the University of Pittsburgh Institutional Biosafety Committee and was performed under BSL2+ conditions. Lentiviral plasmids were packaged in 293FT cells (Invitrogen) using vectors supplied with the ViraPower Expression kit (Invitrogen, San Diego, CA). Viral supernatants were harvested 48 and 72 hr after transient transfection, filtered through 0.45 µM nitrocellulose filters (Millipore, Inc., Bedford, NY) and were used to infect semi-confluent Rat1a fibroblasts [30], [53], [54], in the presence of 8 µg/ml Polybrene (Sigma-Aldrich, St. Louis, MO). After two sequential overnight infections, the Rat1a cells were incubated for an additional 48 hr before being split 1∶3 and selected in the presence of 2 µg/ml of blasticidin (Invitrogen). Blasticidin-resistant clones were then pooled and used in all subsequent studies. We refer to these cell lines according to the identity of the transduced Myc protein (e.g. wtMyc cells, vector control cells, Q131R cells, C133S cells, W135E cells, and F138C cells). Unless otherwise stated, both 293FT cells and Rat1a cells were propagated in Dulbecco's-modified minimal essential medium (D-MEM) containing 10% fetal bovine serum, glutamine, and penicillin plus streptomycin as previously described [53], [55].

### Immunoblotting

Lentiviral-transduced Rat1a cells were grown to approximately 90% confluency under standard conditions and were then harvested with trypsin. After washing twice in PBS, cell pellets were lysed in SDS-PAGE lysis buffer without β- mercaptoethanol and protein concentrations were quantified using a BCA protein determination kit (Pierce, Rockford, IL). 100 µg of total lysate from each cell line was then resolved on a 10% polyacrylamide gel and transferred to a PVDF membrane (Millipore) by semi-dry electroblotting as previously described [30], [53]. Immunoblotting was performed as previously described [42] with a monoclonal antibodies against c-Myc (9E10; Santa Cruz cat. no. sc-40) and β-tubulin (Santa Cruz- cat. no. sc-5274) to serve as a loading control. The blots were developed using a chemiluminesence-based method as previously described [30], [42].

### Apoptosis and Proliferation Assays

For apoptosis assays, Rat1a cells were plated in 12 well tissue culture plates and grown to approximately 80% confluency. They were then washed twice in serum-free D-MEM and incubated in the same medium for the remainder of the study. At the beginning of the study and at various times thereafter, the cells were harvested with trypsin and stained with FITC-Annexin V and propidium iodide using a ApoTarget™ Kit as recommended by the supplier (Invitrogen). Results were expressed as the mean of triplicate assays +/− the standard error (SE). Proliferation assays were performed in D-MEM containing 10% FBS as previously described [30], [53]. The results of proliferation assays were also expressed as the mean of triplicate samples +/− 1 SE. All of the above experiments were repeated at least three times with similar results.

### Measurement of genomic instability

The induction of tetraploidy following the short term exposure to colcemid was performed as previously described [37], [38]. The quantification of ROS was performed as previously described [39] except cells were incubated in 1% serum at 37C for 16 hours prior to staining with 2.5 µM CM-H_2_-DCFDA (Molecular Probes-Invitrogen). Cells were subsequently analyzed by flow cytometry and data is represented as previously described [42]. In brief, the calculation of ROS was derived by first establishing a consistent histogram for control cells stained with CM-H_2_-DCFDA and then subsequently comparing the experimental flow profiles to that control. Samples were run in triplicate and experiments were repeated at least three separate times. Values are expressed as relative mean values ± SE.

### Measurements of OXPHOS and glycolysis

All measurements were performed with a Seahorse Bioscience XF24 Extracellular Flux Analyzer (Billirica, MA). Rat1a cell lines were plated onto Seahorse 24 well plates as recommended by the manufacturer. Unless otherwise stated, the standard Rat1a cell concentration was 20,000/well, which were seeded the day before. Immediately following the addition of fresh medium, basal levels of O_2_ consumption and proton production were first quantified over approximately 20–25 min. The next measurement was performed during the blockade of complex V by 1 µM oligomycin (injection 1). This causes a build-up of protons across the inner mitochondrial matrix with subsequent loss of electron flow. The addition of cyanide *p*-trifluoromethoxy-phenylhydrazone (FCCP) (final concentration 300 nM; injection 2) causes the protons on the outside of the inner membrane to be carried across to the mitochondrial matrix. The addition of 2-deoxyglucose (2-DG) (final concentration 100 mM; injection 3) inhibits glucose uptake into glycolysis and the TCA cycle. Finally, the injection of rotenone (final concentration 1 µM; injection 4) inhibits complex I, leading to cessation of both electron flow and oxygen consumption. Experiments were performed by simultaneously measuring three to five replicates of each cell line. Four of the six cell lines could be measured in a single experiment. Relative effects were expressed as areas under the curve measurements that were generated by the manufacturer's software and used to compare the various cell lines.

### Measurement of cellular transformation

Soft agar assays were used to measure in vitro transformation capacity of Rat1a cells expressing various Myc proteins [12], [30], [56]. Tumor studies were performed by subcutaneously injecting the flanks of nude mice with 5×10^6^ of the indicated Rat1a cell lines [56], [57]. Tumor volumes were calculated using the formula: ½ × L × W × H [58].

### Positron emission tomography (PET)

Tumor-bearing mice were injected with approximately 250 µCi ^18^F-2-deoxyglucose (^18^F-2-DG) via the tail vein twenty minutes prior to imaging with a PET scanner. Mice were anesthetized with isofluorane during the procedure. Standardized uptake values (SUVs) were calculated for each tumor.
